# Multisite randomised controlled trial of a novel dialogical therapy in comparison to treatment as usual in adults with distressing and persistent auditory hallucinations: study protocol for the Talking With Voices (TWV-II) trial

**DOI:** 10.1186/s13063-025-09018-y

**Published:** 2025-10-13

**Authors:** Eleanor Longden, Verity Bell, Emmeline Joyce, Samantha Bowe, Alison Branitsky, Dirk Corstens, Rob Dudley, Richard Emsley, Daniel Freeman, Callum Glen, Amy Hardy, Wendy Jones, Melissa Pyle, Bryony Sheaves, Anthony P. Morrison

**Affiliations:** 1 Division of Psychology and Mental Health; School of Health Sciences: Faculty of Biology, Medicine and Health, Manchester Academic Health Science Centre, The University of Manchester, Manchester, UK; 2 The Psychosis Research Unit, Greater Manchester Mental Health NHS Foundation Trust, Manchester, UK; 3 Complex Trauma and Resilience Research Unit, Greater Manchester Mental Health NHS Foundation Trust, Manchester, UK; 4 GGZ Noord-Holland Noord, Texel/Den Helder, The Netherlands; 5 Gateshead Early Intervention in Psychosis Service, Cumbria, Northumberland, Tyne and Wear NHS Foundation Trust, Gateshead, UK; 6 Department of Psychology, University of York, York, UK; 7 King’s Clinical Trials Unit, King’s College London, London, UK; 8 Department of Biostatistics and Health Informatics, Institute of Psychiatry, Psychology and Neuroscience, King’s College London, London, UK; 9 Department of Experimental Psychology, University of Oxford, Oxford, UK; 10 Oxford Health NHS Foundation Trust, Oxford, UK; 11 Department of Psychology, Institute of Psychiatry, Psychology and Neuroscience, King’s College London, London, UK; 12 South London and Maudsley NHS Foundation Trust, London, UK

**Keywords:** Auditory hallucinations, Psychosocial intervention, Serious mental health problems, Randomised controlled trial, Trauma

## Abstract

**Background:**

Hearing voices (“auditory hallucinations”) is associated with numerous negative outcomes, including hospitalisation, suicidality, and impaired functioning. Currently, the main treatment approaches are antipsychotic medication and cognitive behavioural therapy (CBT), yet both have variable effectiveness and are often unavailable to those without a schizophrenia diagnosis. Furthermore, CBT does not consistently address the role of trauma in voice onset and maintenance. In response to these unmet needs, a feasibility/acceptability trial of a new intervention, Talking With Voices (TWV), was conducted. TWV involves a therapist speaking to the voice(s) while the client repeats its response verbatim, with the aim of promoting recovery and reducing voice-related distress. This prior pilot study (*N* = 50) found excellent feasibility/acceptability data amongst participants with schizophrenia, and signals of positive change in measures of personal recovery and voice relating. The next step is to evaluate treatment mechanisms and clinical efficacy of TWV in a transdiagnostic population.

**Methods:**

We aim to establish TWV’s clinical efficacy in a multisite RCT for adults with serious mental health problems (SMHP) who hear persistent, distressing voices, and to assess whether improved measures of personal recovery and negative voice impact are mediated via key psychological mechanisms (improved relating to, and beliefs about, voices; and reductions in dissociation and negative self-beliefs). We aim to recruit 296 participants from psychiatric services across 4 UK sites (Manchester, London, Newcastle, and Oxford) who will be randomised to either treatment (TWV + treatment as usual [TAU]) or control (TAU only). The primary outcome is total score on the Questionnaire About the Process of Recovery. Secondary outcomes include overall voice severity and other relevant dimensions of voices and trauma sequalae, with mediational and outcome variables collected at baseline, 8 months (post-treatment), and 14 months.

**Discussion:**

The study will investigate the clinical efficacy of a novel intervention deliverable within healthcare services, including hypothesised mechanisms of change to identify key psychological targets for ameliorating distressing voices in a transdiagnostic population. Potential benefits include improving the effectiveness and accessibility of evidence-based psychosocial interventions for SMHP.

**Trial registration:**

ISRCTN, ISRCTN15897915. Registered 13 July 2023.

**Supplementary Information:**

The online version contains supplementary material available at 10.1186/s13063-025-09018-y.

## Introduction

### Background and rationale {6a}

While voice hearing (the perception of speech with no objective source) is a common human experience, distressing voices are often reported by patients with a range of serious mental health problems (SMHP), including psychosis/schizophrenia [1], bipolar disorder [2], depression [2], and borderline personality disorder [3]. Incidence likewise appears high, with a recent survey of 1800 NHS patients diagnosed with non-affective psychosis finding that 48.2% heard voices at least weekly [4]. Correspondingly the impact of voice hearing in these groups can be severe, including increased hospitalisation, suicidality, self-harm, and impaired social and occupational functioning [5–7]. In turn schizophrenia, the condition with which voice hearing is most closely associated, is classed as one of the top 25 causes of disability worldwide and is a significant economic burden in the UK, with total monetary costs estimated as £11.8 billion per year [8] and approximately 222,000 people being treated by the National Health Service (NHS) for schizophrenia and schizophrenia-related disorders at any one time [9]. Strikingly, however, it has been noted that there is little difference in clinical and phenomenological voice characteristics across these different SMHP diagnoses [10], emphasising the need and opportunity for transdiagnostic treatment strategies.

Given the toll exerted by voice hearing, there remains considerable scope for improving clinical care. Antipsychotics are a first-line treatment, yet a proportion of patients respond poorly [11], particularly those with a history of trauma exposure [12], and adverse effects often lead to reduced compliance [13]. Indeed, a meta-analysis of 167 double-blind randomised controlled trials (RCTs) found only 23% of patients with schizophrenia had a “good” response to antipsychotics [14]. Likewise, cognitive behavioural therapy for psychosis (CBTp), the talking therapy recommended by the National Institute of Health and Care Excellence (NICE), is not associated with consistent improvements in voice hearing [15], is not available to those with non-psychosis diagnoses, and may not specifically target voices during treatment. For example, a systematic review of 33 studies indicates only 24.8% of patients exhibit a “much improved” reduction of positive psychotic symptoms, including voices, following CBTp [16]. Both the presence and content of voices also demonstrates strong links with trauma [17–19] yet while the importance of personalised, trauma-informed care forms part of the NHS Long-Term Plan for mental health, this is not something consistently provided by CBTp. It is also clear that disparities exist in receipt of CBTp for those experiencing racial inequalities [20]. As tackling SMHP is a current UK government priority, there is potential for a transdiagnostic psychological treatment to be implemented within the NHS. In turn, this would rationalise training/supervision of staff and permit generalisation of skills across services while avoiding diagnostic “silos” that prevent access. Given urbanicity and social adversity are associated with increased voice hearing prevalence, providing treatments for implementation in groups with high rates of deprivation is also of particular importance, as is equity of access for different racial communities.

Taken together, it is clear distressed voice-hearers are in urgent need of more effective support, particularly evidence-based transdiagnostic strategies that can address the known role of trauma in the onset, maintenance and phenomenology of voices [21]. Specifically, therapies targeting traumatic sequelae which act as mechanisms in voice hearing have the potential to improve outcomes, and our study will focus on the established trauma responses of dissociation, voice relating styles, beliefs about voices and negative self-beliefs, which have been found to mediate the trauma/voice hearing relationship [22–25]. In this regard, patient preference indicates that valued outcomes encompass holistic definitions of personal recovery [26], and research has indicated that the emotional consequences of voice hearing can be strongly associated with such personal recovery [27].

#### Knowledge gaps addressed by the project

Talking With Voices (TWV) is based on a theoretical model of voice hearing which uses direct verbal engagement with the voice(s) by a therapist to instigate a process of reconciliation and integration [28], thereby aiming to resolve trauma-related dissociation, negative self-beliefs, and problematic dynamics in the hearer/voice relationship. Its emphasis on the relational aspects of working with voices, combined with its focus on the associations between voice hearing, adverse life events, and beliefs about oneself, additionally makes it distinct to existing approaches targeting hallucinations, trauma, and/or dissociation [29–33]. Furthermore, TWV is a user-informed approach developed from the work of the Hearing Voices Movement (HVM), an international network of voice-hearers and their allies which has worked since the 1990s to promote more psychosocially focussed, recovery-oriented views of voice hearing [34]. However, despite its international impact, practices developed by the HVM have largely remained unevaluated, mostly due to the primacy it places on personal testimony and an “uneasy relationship” with traditional scientific methodology [34]. The current trial thus presents a valuable opportunity to combine these different traditions and perspectives.

Existing limited data for TWV includes case examples [35–37], a concurrent multiple baseline design case series (*n* = 15) [38], a small RCT (*n* = 12) [39], and our National Institute for Health Research (NIHR) funded feasibility RCT (*n* = 50) [40, 41], all of which provided signals of efficacy with no emergent safety concerns. The latter feasibility/acceptability pilot (outlined below) represents the most comprehensive evidence currently available and, consistent with the Medical Research Council (MRC) framework for developing and evaluating complex interventions, an efficacy evaluation is the next necessary step.

#### Proof-of-concept

TWV adopts a theoretically informed approach to target trauma-related psychological mechanisms in voice hearing, with the aim of improving outcomes, given the known link between trauma and voice phenomenology [17–19]. Dissociation, a psychological response to trauma wherein emotional and cognitive systems become disconnected from one another, is likewise strongly associated with voice hearing [42], and has also been shown to mediate its relationship with trauma [43]. Further, traumatic events are known to have an adverse impact on self-beliefs and relationships with others, which is hypothesised to shape negative voice hearing content/beliefs and how one relates to the voices, which in turn can further exacerbate dissociation [22–24, 44]. Our TWV protocol [28] uses direct verbal engagement from a therapist to instigate a process of reconciliation and integration between hearer and voice, thereby improving connection with emotions, self-concept, and interpersonal relating.

The influence of the HVM on TWV’s development has ensured it was designed to provide a personalised intervention aligned with patient values; for example, being structured around subjective goals [45], holistic engagement with the experience of voice hearing [5], and providing psychosocial support complimentary to medical approaches [46]. Furthermore, it corresponds with several key recommendations made by the International Consortium for Hallucination Research [47] for refining psychological therapies for voice hearing, namely by (1) extending a focus on overall efficacy to understanding specific therapeutic processes, (2) a better targeting of psychological processes associated with voice hearing, such as trauma, cognitive mechanisms, and personal recovery, and (3) using focused measurement of the intended outcomes of therapy.

#### Feasibility study

The current research plan is an expansion of the TWV pilot trial, which compared TWV + treatment as usual (TAU) with TAU alone amongst adults with a diagnosis of schizophrenia spectrum disorders [40, 41]. Owing to its co-produced nature, the intervention itself was not originally developed within the (MRC) framework for the development of complex interventions [48]. However, subsequent pilot work, including manualisation of the therapy, was conducted according to MRC guidelines to establish the feasibility and acceptability of delivering TWV within the infrastructure of the NHS (including identifying key uncertainties and potential refinements). Consequently, the research programme is positioned to progress to clinical evaluation.

The TWV pilot recruited to target (50/50; 100%), with excellent rates of treatment uptake (21/24 [87.5%] participants receiving ≥ 8 sessions) and retention in the trial (40/50 [80%] participants at 6-month follow-up). Withdrawals were likewise low, with only 1 participant withdrawing from the therapy arm and 2 from TAU. Although not powered to detect treatment effects, a statistically significant increase in perceived benevolence of the voice was observed (− 3.93(SE 1.63); 95%CI − 7.27, − 0.58; *p* = 0.02) amongst participants receiving TWV, as well as a suggestion of increased personal recovery (− 6.94(SE 4.41); 95%CI − 16.00, 2.12; *p* = 0.13) and reduced dissociation (7.22(SE 7.17); 95%CI − 7.65, 22.08; *p* = 0.33). In this regard our proposed primary outcome measure for the current trial, the Questionnaire About the Process of Recovery (QPR) [49], resulted in a between-group standard effect size of 0.7. In turn, there was a lower rate of serious adverse events (SAEs), including hospital admissions, in the therapy group relative to TAU, none of which were deemed trial-related by the combined Trial Steering Committee (TSC) and Data Monitoring and Ethics Committee (DMEC).

Nested qualitative studies with both trial participants [50] and therapists [51] indicated several features of the intervention that were positively received. For participants, this included the opportunity to develop strategies to cope with hostile voices, the experience of a close therapeutic alliance, gaining new perspectives on voice utterances, discovering links between voice content and negative life events, and learning to relate to voices in more constructive ways. In this regard, withdrawal rates can be in the region of 18% for therapies which include some element of aversive exposure, including treatments for post-traumatic stress [52] and direct work with voices [29], and between 20 and 24.5% in trauma-focussed therapies for psychosis patients [30]. However, only 3/24 participants (12.5%) attended less than the 8 sessions constituting a therapeutic “dose” of TWV, with only one participant (4.2%) dropping out of the therapy arm of the trial. Planned refinements to both recruitment procedures and treatment protocols have been made as a result of this analysis, including extending the therapy window and clarifying therapy aims in study informational materials. For therapists, in turn, TWV was felt to be a unique intervention that permitted an exploration of clients’ voice hearing experiences that was unavailable in other therapeutic models. Numerous examples were provided of acquiring and implementing new knowledge while augmenting/transferring existing skills, with therapists additionally referring to positive experiences of integrating both recruitment and therapy delivery within participants’ existing healthcare teams. Several recommendations for therapist training and supervision were likewise derived from this work. Further, the results confirmed that CBTp therapists already experienced in working with voice-hearers were able to deliver the intervention without time-intensive training, which has positive implications for scalability.

Taken together, the pilot suggests that a larger trial of TWV will be acceptable and feasible to staff and service users within the NHS, and that the expansion of the intervention into a transdiagnostic population may be of clinical benefit for those troubled by persistent, distressing voices. In turn, analyses of both quantitative and qualitative data indicate long-term potential for enhancing service provision (a transferrable clinical model that utilises existing skills) and developing patient benefit (improved quality of life, improved rates of recovery, reduction in distressing voices).

### Objectives {7}

The study aims to address two principal research questions: (1) Is the psychological intervention TWV + TAU effective in improving personal recovery compared to TAU alone at post-treatment (8-month follow-up) in adults with SMHP who hear persistent, distressing voices, and do these benefits endure at 14-month follow-up? and (2) Are any identified treatment effects of TWV on recovery mediated by key mechanisms, specifically: improved relating with, and appraisals about, voices; reduced dissociation; and a reduction in negative self-beliefs at post-treatment, and do these benefits endure at 14-month follow-up? In addition to quantitative outcomes, a series of nested qualitative sub-studies will also be conducted to gain further data into subjective patient and therapist experiences of receiving and delivering the intervention, explore any perceived changes and mechanisms of change, and examine barriers and facilitators to implementation (please see “Plans for assessment and collection of outcomes {18a}”).

Specific aims and hypotheses are as follows:

#### Clinical efficacy aims

1. To establish the efficacy of TWV + TAU in improving measures of personal recovery compared to TAU alone when delivered to adults with SMHP who hear persistent, distressing voices at post-treatment.
2. To establish the efficacy of TWV + TAU in reducing the impact of distressing voices compared to TAU alone at post-treatment.
3. To establish the efficacy of TWV + TAU in reducing negative appraisals of voices and increasing positive appraisals of voices and helpful/functional responses towards voices compared to TAU alone at post-treatment.
4. To determine whether positive effects of TWV are detectable over a 14-month follow-up period.

#### Primary clinical efficacy hypothesis

TWV + TAU will result in improved measures of personal recovery at post-treatment compared to TAU alone.

#### Secondary clinical efficacy hypotheses

1. TWV + TAU will lead to improvement in distressing voices at post-treatment compared to TAU alone.
2. TWV + TAU will lead to a reduction in negative appraisals of voices and increased positive appraisals of voices and helpful/functional responses towards voices at post-treatment compared to TAU alone.
3. TWV + TAU will result in improved measures of personal recovery and voice-related measures at 14-month follow-up compared to TAU alone.

#### Mechanistic aim

To examine the extent to which TWV + TAU impacts on measures of personal recovery via reductions in trauma-related psychological processes (dissociation and negative self-beliefs), and improvements in positive beliefs about voices and assertive relating skills with voices, at post-treatment and 14-month follow-up.

#### Mechanistic hypotheses

1. TWV + TAU will lead to reductions in dissociative symptoms and negative self-beliefs, and improvements in positive beliefs about voices and assertive relating skills with voices at post-treatment and at 14-month follow-up.
2. The mechanisms by which TWV + TAU leads to improvements in personal recovery is due to a reduction in dissociative symptoms and negative self-beliefs, and improvements in positive beliefs about voices and assertive relating skills with voices at post-treatment and at 14-month follow-up.

#### Research objectives

We intend to recruit 296 adults with SMHP who hear persistent and distressing voices from NHS mental health services across 4 UK sites (Greater Manchester, London, Newcastle, and Oxford). Eligible participants will be randomised to either the treatment arm (TWV + TAU) or control arm (TAU alone) across 8 months. Outcome data will be collected at baseline, at 8 months (post-treatment) and at 14 months follow-up (for approximately 207 participants, owing to the variable follow-up period: please see “Plans for assessment and collection of outcomes {18a}”).

##### Trial design {8}

TWV-II is an assessor-blinded, multisite RCT assessing the efficacy and mechanisms of a psychological therapy (TWV) for adults with SMHP who hear persistent and distressing voices. The 2 parallel arms will use a superiority hypothesis framework to compare TWV + TAU (treatment condition) to TAU alone (control condition). Assessment of outcome and mediational variables will take place at baseline, at 8 months, and at 14 months. In addition to TAU, participants randomised to the treatment arm will receive up to 26 weekly sessions of TWV of approximately 1 h duration, with an option for up to 4 booster sessions. Independent, concealed randomisation at a ratio of 1:1 is performed via a web-based system using random permuted blocks, stratified by site and diagnosis, by King’s Clinical Trials Unit ([KCTU] UKCRC registration 053). The protocol is reported according to SPIRIT guidelines (53).

## Methods: participants, interventions, and outcomes

### Study setting {9}

The study is conducted in NHS community-based, secondary care mental health services within four sites, and five NHS Trusts, within the UK: Berkshire Healthcare NHS Foundation Trust (a Participant Identification Centre for the Oxford site); Cumbria, Northumberland, Tyne and Wear NHS Foundation Trust; Greater Manchester Mental Health NHS Foundation Trust [GMMH]; Oxford Health NHS Foundation Trust; and South London and Maudsley NHS Foundation Trust. Both assessments and intervention delivery take place in either participants’ homes or NHS premises.

### Eligibility criteria {10}

The study population are adult users of mental health services with SMHP who hear persistent, distressing voices.

Our inclusion criteria are as follows:

1. Aged ≥ 16 years.
2. Heard voices for at least a year.
3. Scoring ≥ 1 on item 8 of the Psychotic Symptom Rating Scales–Auditory Hallucinations Subscale (PSYRATS-AH) [54].
4. Able to provide written informed consent.
5. Actively help-seeking in relation to distressing voices.
6. In contact with mental health services for ≥ 6 months.
7. Willing and able to communicate with their voices and relay what the voices say to a therapist.
8. Hear voices that are sufficiently personified to engage in dialogical work.^1^

Our exclusion criteria are as follows:

1. At immediate risk of harm to self or others.
2. Currently receiving structured, individual psychological therapy.
3. Non-English speaking.
4. Primary diagnosis of alcohol/substance dependence or autism spectrum disorder.
5. Moderate/severe learning disability.
6. Organic cause for voices.
7. Homeless/of no fixed abode.

### Who will take informed consent? {26a}

Informed consent is obtained across all sites by research assistants (RA) trained in Good Clinical Practice (GCP) who are supervised by their site’s Principal Investigator (PI) and the trial manager, and in a manner consistent with the National Research Ethics Service [55]. Prior to taking written informed consent, all potential participants are provided with the study’s participant information sheet (PIS) and given at least 24 h to consider the information and receive answers to any questions they may have before consenting. Recording informed consent in writing via a wet-ink signature is prioritised from both participant and researcher. However, in the event of it being unfeasible to seek written consent (e.g. where COVID-19 restrictions apply), consent is taken remotely via telephone or MS Teams following sponsor and Research Ethics Committee (REC)-approved processes (specifically, an audio recording of the consent meeting is retained in the electronic Investigator Site File (ISF) whereby the participant states their name and date of consent then verbally confirms their agreement with each statement from the consent form as read aloud by the researcher). The RA then adds a wet-ink signature to the remote consent form and, where possible, a wet-ink signature is sought from the participant at a subsequent meeting. Copies of consent forms are provided to all participants.

Consent for trial participants to take part in the nested qualitative studies will be obtained by the qualitative researcher before the interview begins for permission for audio-recording of interviews and for anonymised direct quotes to be used in publications. Therapists’ consent to take part in a focus group will be obtained by the qualitative researcher, likewise prior to the group convening.

### Additional consent provisions for collection and use of participant data and biological specimens {26b}

All trial participants are asked if they would consent to their data being used to support future research and shared anonymously with other researchers; if they agree to their research assessments being recorded; and, in the event of receiving the intervention, if they agree to recording their therapy sessions, and/or would consent to be contacted for an interview about their experiences of the therapy. It is made clear that these items are optional and declining consent does not prohibit participation in the trial or have any effect on existing care. Participants taking part in a qualitative interview can additionally request to withdraw their data at the point of the interview, or within 3 weeks thereafter. However, it will not be possible for participants to withdraw once their transcribed interview has been anonymised and forms part of the dataset.

## Interventions

### Explanation for the choice of comparators {6b}

Given that different psychosocial interventions are recommended in NICE guidelines for different diagnostic groups, no single active comparator would be suitable and the control condition for the trial, therefore, is TAU. In the UK, TAU for SMHP is based on the Care Programme Approach and typically includes psychiatric medication, assignment of community-based health and social care staff, care coordination, access to rehabilitative services, and outpatient care. With the exception of emergent risk issues, TAU alone will not involve liaison between researchers and the participants’ healthcare teams, and referrers for participants in either arm will not be requested to withhold any treatment throughout the duration of the trial. In this respect, while receipt of psychological therapy is an exclusion criterion at the point of referral, it is likely that some TAU participants will receive such support during the treatment or follow-up windows.

### Intervention description {11a}

The two parallel arms of the trial are a psychological intervention (TWV) + TAU (treatment condition) vs. TAU alone (control condition).

#### Treatment condition

The trial has utilised a revised version of the treatment manual created and refined during the TWV pilot [28]. Therapy proceeds across four stages (Psychoeducation, Formulation, Dialogue, Consolidation) and, in brief, employs individualised formulations to identify key psychosocial conflicts associated with voices and determine targeted treatment strategies and shared goals for relational Change via dialogue. An 8-month treatment window permits ≤ 26 sessions, with an option for up to 4 booster sessions to consolidate therapeutic gains. A range of interventions with associated milestones are delivered within the treatment timeframe (Table 1).

**Table 1:** Therapy phases and associated milestones for Talking With Voices

Phases	Approximate session number	Therapy milestones
Engagement & psychosocial education	1–4	Establishing client contact and explaining intervention Discussing experiences of, and beliefs about, hearing voices Normalising and destigmatising voice-hearing Psychosocial education focusing on the relationship between voice-hearing, life circumstances and negative emotions Establishing an alliance with the voices Commencing development of self-care and coping/grounding skills Introducing between-session tasks Preparation for future stages of therapy
Assessment & formulation	5–9	Developing a construct that encompasses all the voices a person hears
		Based on the construct, have a shared understanding of (1) who or what the voices represent, and (2) what problems the voices represent Continuing to collaboratively set between-session tasks
	10–11	Make a report of the construct and have a conversation about the report
Dialogical work	12–13	Reiterating therapy aims
		Planning which voices to speak with, the issues to explore, and gaining voices’ permission Developing acceptable shared goals for the dialogue Pre-agreeing a signal (“panic button” metaphor) for ending the dialogue and establishing the voice hearers’ capacity to take control again when asked Focusing on the reactions of the voice(s): repeatedly asking how they are feeling about what is happening; asking permission; acknowledging their role; making empowering comments; setting limits; respectful language towards the voices, including replacing derogatory names Identifying an ally in the voice hearer’s life to involve in Stage 4 Continuing to collaboratively set between-session tasks
	14–23	Achieving a direct dialogue with the voice
		Collaborative evaluation and appraisal of the conversation Planning future conversations and between-session tasks Establishing boundaries for the voice via “time-sharing”. If applicable, developing additional strategies to counter voices’ perceived omnipotence and/or to become more assertive with them Encouraging voices to use therapy sessions as a space to express their own frustrations, rather than harassing the client during the week Developing short replies/mantras that the client can use between sessions in response to the voices’ concerns If desired/available, assist client to access a local Hearing Voices Network peer-support group
Evaluation & consolidation	24–26	Create a collaborative summary of (1) what was achieved during therapy, (2) ways of implementing the strategies learned during therapy and identify strategies/goals for the future (e.g. continue time sharing, using respectful language to the voice, not obeying commands, self-soothing), and (3) planning for future difficulties Handover session with identified family member and/or healthcare worker for support to take the work forward and (if desired/available) provide signposting to relevant local services

The manual adheres to general best-practice principles for psychological therapy with psychosis patients, including building collaborative relationships, developing shared goals, using inclusive language, validating individual experiences, and providing hope that recovery is possible [56]. In turn, these principles underpin many of the specific values of TWV, which can be summarised as follows:

1. *A normalising approach*: Voice hearing is recognised as a common human experience that may cause distress but from which many people recover. Consistent with the ethos of the HVM, the concept of recovery is not solely defined by cessation of clinical symptoms but rather in helping to reduce distress and promoting positive goals, with full recognition that individuals can live fulfilling lives as voice-hearers.
2. *A user-led intervention*: clients have a central role in determining the pace and goals of therapy and identifying the most useful strategies to cope with their experiences.
3. *A subjective interpretative framework*: therapists respect their clients’ explanatory framework for understanding voices (e.g. trauma-based, spiritual, cultural) without insisting a clinical perspective is the correct one.
4. *Conceptualising voices as representing parts of the self*: voices are considered a dissociative phenomenon which may often originate from traumatic events and/or reflect overwhelming emotion along with negative beliefs about oneself, other people, and the world. Correspondingly, voice content is seen as meaningful in the sense of drawing attention to unresolved distress.
5. *Facilitating a more peaceful hearer-voice relationship*: in signposting emotional vulnerabilities, voices can be seen as performing a “protective” role in the sense that features like persecution or aggression may be masks for unresolved pain. Because attempts to supress the voice will also suppress the emotions and conflicts which they embody, a complementary goal is therefore to help the voice communicate its purpose and needs in ways that are more constructive and respectful of the hearer.

#### Training and supervision

Trial therapists require accredited therapeutic training, either as a Clinical Psychologist or training for a specific therapeutic modality (such as CBTp), and are expected to demonstrate substantive prior experience of facilitating therapeutic interventions in health and social care settings, offering trauma informed therapeutic work, and supporting individuals who experience SMHP. They have often had experience of working in Early Intervention in Psychosis (EIP services) and Community Mental Health Teams (CMHTs), which are the services where many trial participants are recruited from. To deliver the intervention, therapists receive an initial 3-to-5-day training package (with an additional “top-up session” provided 10 months into the delivery window) and attend weekly group supervision delivered by both a clinician and lived experience expert. They are additionally provided with a therapy manual and access to approved therapy resources, with adherence to the therapeutic model monitored across sites by the trial’s clinical co-lead.

### Criteria for discontinuing or modifying allocated interventions {11b}

Participants who lose capacity to consent will be withdrawn from research procedures associated with the study, and participants are likewise informed of their right to withdraw from the research at any time without giving a reason and without their care being affected. If a participant does wish to withdraw they are provided with options, including full trial withdrawal, or partial withdrawal (i.e. withdrawing from therapy but continuing with research assessments, thus retaining a greater proportion of follow-up data). Participants allocated to the intervention arm are further provided with the option to take a break in therapy, or to change the focus of the sessions. Prior to giving informed consent, participants are made aware that should they choose to withdraw from all research procedures we will not collect any further outcome data but will retain the data we have collected up until the point of withdrawal. Any participants who withdraw will not be replaced. Procedures for managing withdrawals from the study are outlined in a trial-specific standard operating procedure (SOP), adhered to by all staff working on the project.

### Strategies to improve adherence to interventions {11c}

Fidelity to the TWV approach is monitored during weekly 90 to 120-min group supervision sessions for each site, including the use of audio recordings from TWV sessions (where participants have provided consent) during which therapists receive feedback from lived experience expert and clinical expert supervisors, as well as their therapist peers. Adherence is monitored by the clinical co-lead using an adherence database which collects information on therapy milestones embedded in the treatment manual, the number of sessions attended, and which therapy phase the session focused on. The adherence database further monitors the specific strategies utilised within that session, and whether between-session tasks for both participants and therapists have been set.

### Relevant concomitant care permitted or prohibited during the trial {11d}

At the point of consent participants have been under the care of mental health services for at least 6 months and will have access to the type of services already offered as part of TAU. Typically, these will be treatments relevant for the participant’s mental health condition as outlined in NICE guidelines, and no care will be withheld or prohibited during trial participation.

### Provisions for post-trial care {30}

Formal post-trial care is not offered by the research team. Upon exiting the trial, participants will retain existing access to TAU and be provided with a crisis card detailing national statutory (NHS) and non-statutory (voluntary) sector helpline services. In the event of a participant being harmed during the research, they may have grounds for legal action for compensation against the sponsor NHS Trust (GMMH NHS Foundation Trust). All participants are further able to make a complaint about the study via the usual NHS channels.

### Outcomes {12}

Efficacy outcomes will assess overall personal recovery and the impact/severity of voices, with additional clinically relevant outcomes of voice-related and trauma-related phenomenology and negative self-beliefs. Summary statistics (mean and median) will be reported for primary, secondary, and mechanistic outcomes, overall and by arm, at each timepoint that they are collected.

The primary outcome for the study is personal recovery at 8 months post-randomisation (end of therapy) as measured by the QPR, a self-report measure designed in collaboration with service users to assess personal recovery from psychosis. The total QPR score will be the specific measurement variable, using an analysis metric of change from baseline. The secondary outcomes will assess overall voice severity and other relevant dimensions of psychiatric distress and trauma sequalae that it is anticipated the therapy will affect. Specifically, we will assess voice phenomenology and impact, acceptance-based attitudes and actions towards voices, presence and impact of non-auditory hallucinations, severity of trauma-related symptoms, connections between adverse life events and voice hearing symptoms, and dissociation. Mechanistic outcomes will also be assessed, namely negative beliefs about the self, depersonalisation/derealisation, interactions with voices, and assertive responding to voices. Total or sub-scale scores for each questionnaire/interview will act as the measurement variable. Please see “Data collection and management” for a full list and description of measures.

To appropriately characterise the sample, demographic variables (age, gender, ethnicity, religion, highest level of education, employment status, marital status, living arrangements) and clinical variables (current psychiatric diagnosis, duration of voice hearing, current psychiatric medication use, historic engagement with psychological therapy) will be collected at baseline using a customised form. Lifetime trauma exposure will also be measured at baseline via a validated instrument (see “Data collection and management”) and will include type of trauma experienced, timing, and multiple exposure for all traumatic experiences.

The experience of receiving and delivering the intervention will be assessed as a further secondary outcome in both the treatment group and amongst trial therapists. Specifically, these three nested sub-studies will explore (1) the influence of TWV on one’s mental health and recovery, including the impact of its hypothesised mechanisms of action, (2) the influence of racial identity on receiving and engaging with therapy, and (3) perspectives on delivery and implementation of TWV within healthcare services.

### Participant timeline {13}

A schedule for participant enrolment, intervention, and assessment is provided in Table 2. Participant movement throughout the study will be documented at each stage, including all withdrawals and reasons for declining to participate. Qualitative interviews with participants will take place after the 8-month treatment window (including any booster sessions) has passed and their 8-month research assessment is completed, and therapists will be approached to take part in a focus group with trial colleagues at their site towards the end of the intervention window (approximately October 2025).

**Table 2:** Schedule of enrolment, interventions, and assessments

**TIMEPOINT**	**Enrolment** **-t1**	**Allocation** **0**	**Post allocation**					
			**Months 1–3**	**Month 4**	**Months 5*–*7**	**Month 8**	**Month 11**	**Month 14**
**ENROLMENT**								
Informed consent	X							
Baseline assessment	X							
Allocation		X						
**INTERVENTIONS**								
TWV	X	X	X	X	X	X	X	X
TAU	X	X	X	X	X	X	X	X
**ASSESSMENTS**								
Demographics form	X							
PSYRATS-AH	X					X		X
QPR	X					X		X
TVAQ	X					X		X
Approve—Voices	X					X		X
BAVQ-R	X					X		X
PSYRATS-MMH	X					X		X
TALE	X							
PCL-5	X					X		X
BCSS	X					X		X
DES-II	X					X		X
VAAS-12	X					X		X
Treatment documentation form						X		X
Trial evaluation form*						X		X
Qualitative interviews (participants and therapists)						X	X	X
Adverse events**						X		X
Thank you card				X			X	X

*PSYRATS-AH* The Psychotic Symptom Rating Scales-Auditory Hallucinations [54], *QPR* The Questionnaire About the Process of Recovery [49], *TVAQ* Trauma Voice Associations Questionnaire [57], *Approve-Voices* the Approve-Voices questionnaire [58], *BAVQ-R* The Revised Beliefs About Voices Questionnaire [59], *PSYRATS-MMH* The Psychotic Symptom Rating Scales-Multimodal Hallucinations (unpublished measure), *TALE* Trauma And Life Events Checklist [60], *PCL-5* PTSD Checklist for DSM-5 [61], *BCSS* Brief Core Schema Scale [62], *DES-II* The Dissociative Experiences Scale-II [63], *VAAS-12* Voices Acceptance and Action Scale - Brief Version [64]

^*^The trial evaluation form is offered to participants at trial exit (i.e. at either the 8-month or 14-month follow-up assessment, depending on the time at which they were enrolled in the trial)

^**^Adverse events will be monitored at each time point, and at every intervention session for the TWV group

### Sample size {14}

The trial is a partially nested design, with clustering due to therapists in the intervention arm and each participant in the control arm considered as a cluster of size 1. We allow for 14 therapists over the course of the trial, with an ICC = 0.02, each therapist seeing an average of 9 participants and variation in the cluster size of 9 (assuming the cluster membership follows a Poisson process). To achieve 90% power to detect a between-group standard effect size (SES) of 0.4 at 8 months on the primary outcome measure, with 5% 2-sided significance level, and assuming a conservative correlation of 0.4 between the respective baseline and 8-month scores and 1:1 allocation ratio, we require 252 participants with outcome data in the analysis set. Allowing for a conservative 15% attrition (attrition was 10% in our pilot trial) requires 296 participants to be recruited. A recent study used an anchor-based method to establish the minimum important difference for the QPR and suggested that a difference of 4–5 points is a worthwhile target difference [65]. Using a difference of 4.5 points, with a standard deviation of 11.5 (based on QPR scores from several of our SMHP trials) this equates to an SES of 0.4, as above. In our pilot trial, we observed an SES of 0.7.

For the nested qualitative studies, we will seek to recruit two groups of up to 25 (i.e. up to 50 total participants) with therapy recipients, for an anticipated final sample size of ≥ 10 for each study. Purposive sampling will be employed for each qualitative study with trial participants, and it is anticipated that the final sample will be representative and include variance on key variables (e.g. therapy engagement, site, age, gender, ethnicity). All therapists currently working on the trial will also be approached for inclusion in the third qualitative study, for an anticipated final sample of 10–16. On the basis of previous work, we expect both participant and therapist samples to be sufficient for achieving thematic saturation (i.e. the point at which no new categories emerge).

### Recruitment {15}

A variety of methods are used to raise awareness of the trial and provide maximum engagement of clinical services and outreach to all potentially eligible service users. Specifically, PIs lead the development of recruitment strategies in each site to align with local services, geography, utilise existing clinical research connections, and support engagement between the study and clinical teams. At the commencement of the trial, a launch event for the trial sites was hosted by the co-Chief Investigator (CI) and trial manager, with RAs also arranging liaison presentations at local NHS CMHT and EIP services to promote the trial, share information of who may be eligible, and advise on how to make a referral. Where possible, a trial therapist also attended these service presentations to answer intervention specific questions. A “re-launch” event of the same format also took place 13 months into the recruitment window to facilitate continued awareness.

RAs establish and maintain relationships with clinical services by arranging times to visit to share study materials and to sit within services to coordinate with potential referrers. In this regard, most eligible participants are identified by NHS professionals in mental health care teams who have established relationships with service users, such as care co-ordinators, although individuals may also self-refer by contacting the study team directly. Eligible participants are further identified by Research Delivery Network (RDN) staff using NHS Trust approved research screening processes. In line with these processes, and where sufficient approvals are in place, RDN staff and trial RAs can also contribute to screening of potential participants if they have been delegated to do so by service team leads. Regular site meetings are used to monitor recruitment methods and identify ways to diversify recruitment, such as developing connections with third sector organisations (e.g. Mind, hearing voices self-help groups). In addition, site meetings are also used to develop strategies for promoting recruitment within services and localities that are typically under-served, as well as engaging groups who tend to be under-represented in psychological research.

Referring healthcare staff are asked to discuss the study with service users on their caseloads who meet preliminary inclusion criteria and, in the event of potential participation, obtain verbal consent to be contacted by an RA. This initial contact, usually a telephone call, aims to introduce the study, answer individual questions, and explore any concerns or barriers the person may have about recruitment. Additionally, if the potential participant is comfortable to do so, the RA asks them about their voice-hearing experiences to ensure that they meet trial inclusion criteria, with the rationale of avoiding the burden of a baseline assessment if they are ineligible at the point of referral (e.g. confirming they have heard voices for at least a year, that they are distressed by their voices and would like help with them, and exploring the extent to which their voices are personified and dialogical). If potential participants remain interested after this preliminary call, RAs offer to send out the Participant Information Sheet (PIS) if this has not already been received and invite them to arrange an assessment appointment. In the event of intervention-specific questions, an option is provided to speak with one of the trial therapists. In the case of self-referrals, RAs will also request permission to contact a named healthcare provider to ascertain eligibility and other relevant referral details.

As part of our recruitment strategy, we utilise existing work from the TWV feasibility trial to ensure the study continues to reach under-served groups. Every person eligible to participate is offered the same opportunity regardless of protected characteristics, and recruitment is monitored in relation to the population characteristics of the study sample within our TSC and DMEC, as well as during site and Trial Management Group (TMG) meetings. Due to a lack of validated outcome tools in non-English languages, as well as the ethical and clinical restrictions of delivering the intervention via interpreters, we are unable to include participants in the trial who do not have sufficient command of English to complete assessment measures and/or engage with therapy (specifically, the feasibility study was not tested in non-English speaking participants, and the challenges this would entail when delivering TWV do not meet British Psychological Society Best Practice Guidelines when working with interpreters [66]). However, this does not exclude people with English as a second language, and we take every measure possible within the protocol and funding arrangements to engage this group; for example, where spoken word is more accessible than written, we would provide materials in the former. Further, where possible within existing funding and resources, we intend to conduct pilot work in which participants excluded due to language barriers would receive the therapy without being randomised, and whose enrolment would not contribute to the overall study recruitment number.

For the first two nested qualitative studies, participants will be recruited via their therapists telling them about the studies and asking if they may be interested in taking part once their treatment window has ended. Participants will be informed that this is optional and separate to their involvement in the trial and therapy, and it will not affect their trial participation or usual mental healthcare if they decline an interview. Participants’ therapists will initially seek permission to pass each participant’s details onto the qualitative researcher(s); however, we may also seek to contact participants whose treatment windows have already passed to broaden the potential sample. In these cases, a member of the trial team will contact the participant initially to seek permission to pass their details onto the qualitative researcher(s). Trial therapists will be informed about the opportunity to take part in the focus group during their clinical supervision sessions and/or over email, and likewise informed that a choice to participate will have no effect on their trial involvement.

## Assignment of interventions: allocation

### Sequence generation {16a}

The randomisation system was created in collaboration with the trial analysts and the CIs and will be maintained by the KCTU for the duration of the project, hosted on a dedicated server within King’s College London (KCL). Randomisation is at the level of the individual, independent, and concealed using the method of random permuted blocks which are unknown to the study team. It is stratified by site and diagnosis.

### Concealment mechanism {16b}

After obtaining written informed consent, completing the baseline assessment measures, and confirming eligibility within the trial team, RAs contact participants to inform them of the outcome. RAs then randomise participants within 2 working days using a trial-specific web-based portal developed and hosted by the KCTU.

### Implementation {16c}

Following randomisation, allocations are made known by email to the trial therapists, the trial manager (to monitor adherence to the randomisation algorithm), the trial administrators (who are delegated to post allocation letters), and the PIs and CIs (to ensure oversight). The allocation is also made known to participants by phone call and follow-up letter from an unblinded staff member, and to relevant individuals from their healthcare team.

## Assignment of interventions: blinding

### Who will be blinded {17a}

Talking With Voices is a single-blind (assessor) RCT. Blinding of the allocation code will be maintained for RAs until all outcome measures for all participants have been collected, and is managed using a range of measures, such as separate offices for therapists and RAs, protocols for answering telephones (including reminders for participants, family members, and clinicians about the blind), protocols for message taking and secretarial support, separate diaries and pigeonholes, and data file security using passwords and encryption of randomisation information. Further, all qualitative interviews with trial participants will occur at a timepoint away from a standard follow-up point and will be overseen by the trial manager to ensure participants and the RAs are not in contact.

The senior trial statistician will be unaware of individual participants’ random allocations or group-level summary data split by arm throughout the course of the trial. The trial statistician will be blinded during the drafting of the Statistical Analysis Plan (SAP) and become unblinded to the individual participant and group-level summary data at the preparation of the first closed DMEC report (approximately 1 year after first participant recruitment). The primary database will also not include any information about allocation, which is contained in a separate system to protect against accidental unblinding, with a secondary database maintained for entering unblinded information (e.g. adverse events, therapy details) which blinded researchers do not have access to. Final analyses will be carried out by the trial statistician and overseen by the senior trial statistician, with investigators and the research team blind to group-level summary data split by arm throughout the course of the trial. A SOP for maintaining, recording, and managing blinding has been developed to outline these procedures, which will be made available to the oversight committees on request and has been received by trial staff as confirmation they understand and will comply with the protocols.

Staff are required to report all blind breaks, which are recorded by the trial manager and reviewed by the CIs for any patterns in unblinding. The DMEC and TSC also monitor unblindings and implement corrective action if necessary. There are only two follow-ups scheduled (at 8 months and 14 months, both after end of treatment), which further reduce the risk of blind breaks by removing the opportunity for therapists and RAs to cross paths while visiting participants at their homes and/or when communicating with participants to arrange visits. All letters to participants and clinicians contain a standardised statement about the need to maintain the single blinding process. In the event of blind breaks, we aim to identify independent assessors to complete subsequent follow-ups (subject to any threats to participant engagement with follow-up). If a change of assessor is not possible, the assessment is audio-recorded with the participant’s permission and another assessor co-rates the PSYRATS-AH and Psychotic Symptoms Rating Scale—Multimodal Hallucinations ([PSYRATS-MMH]: the only assessment measures which are interviewer-rated, rather than participant-rated). Scores are then compared across the blinded and unblinded RAs, with any discrepancies taken to the trial manager for discussion and resolution.

### Procedure for unblinding if needed {17b}

The blind will only be deliberately broken in very exceptional circumstances in which clinical duty of care or participant rights overrule the rationale for the blind. Any deliberate blind breaks, and the procedure for breaking the blind, will be the decision of the trial manager and CIs on a case-by-case basis.

## Data collection and management

### Plans for assessment and collection of outcomes {18a}

After recruitment and baseline assessments are concluded, a follow-up assessment will take place at 8 months post-randomisation (end of treatment). Additional follow-up assessments will be performed at 14 months post-randomisation, dependent upon when participants were recruited into the trial (thus, the total follow-up period will vary from 14 to 8 months, maximising the recruitment period and providing best value for money). In view of this, we anticipate 14-month follow-up data on approximately the first 207 participants.

#### Primary outcome

The primary outcome will be the total score on the 15-item QPR at 8 months, using an analysis metric of mean change from baseline.[49] The QPR was developed in collaboration with service users to assess personal recovery from psychosis, containing items that were initially derived from qualitative interviews about this topic. It has excellent reliability, validity, and sensitivity to change and is nationally adopted as a Patient Recorded Outcome Measure for evaluation of early intervention for psychosis services, forming part of the Mental Health Services Data Set. Patients consistently prioritise personal recovery over specific symptom change [67] and the QPR has been cited [68] as the only measure of recovery that directly maps onto all 5 processes of the influential CHIME framework of personal recovery [69].

#### Secondary outcomes

Secondary outcomes will assess overall voice severity and other relevant dimensions of psychiatric distress and trauma sequalae, assessed as mean Changes from baseline at 8 months (primary endpoint) and 14 months (secondary endpoint). With the exception of the unpublished PSYRATS-MMH, all are standardised semi-structured interviews and questionnaires with demonstrated reliability and validity.

1. The PSYRATS-AH [54], an interviewer-rated measure that assesses voices across 11 domains of phenomenology and impact.
2. The Voices Acceptance and Action Scale - Brief Version (VAAS-12) [64], a 12-item measure designed to assess acceptance-based attitudes and actions in relation to auditory and command hallucinations.
3. The PSYRATS-MMH, an unpublished scale adapted from PSYRATS-AH for assessing the presence and impact of non-auditory hallucinations.
4. The PTSD Checklist for DSM-5 (PCL-5) [61], a 20-item self-report measure that assesses the severity of a range of trauma-related symptoms.
5. The Trauma Voice Associations Questionnaire (TVAQ) [57], a 16-item inventory which assesses connections between adverse life events and voice hearing experiences.
6. The Dissociative Experiences Scale-II (DES-II) [63], a 28-item measure assessing the daily frequency of dissociation across the domains of absorption, depersonalisation/derealisation, and dissociative amnesia.

The proposed mechanisms of action for TWV will also be measured with the following instruments:

1. Negative beliefs about the self will be assessed with The Brief Core Schema Scale (BCSS) [62], a 12-item self-report questionnaire.
2. Dissociative experiences will be measured with the depersonalisation/derealisation subscale of the DES-II [63], which contains 6 items scored for daily frequency.
3. Interactions with one’s voices will be assessed with the Beliefs About Voices Questionnaire – Revised (BAVQ-R) [59], a 35-item measure of beliefs about auditory hallucinations and emotional and behavioural reactions to them.
4. Assertiveness in response to one’s voices will be assessed using the 15-item Approve – Voices Questionnaire [58].

Additional pharmacological and psychological treatments for mental health concerns will be monitored in both arms using a treatment documentation sheet, with feedback about the trial and intervention, including potential adverse effects of participation, collected via a self-report “Learning From You” questionnaire offered to participants on exit from the study following the 8- or 14-month assessment. Copies of both these measures are available from the corresponding author on request. Adverse events (AEs) and SAEs will also be assessed separately (for details, please see “Adverse event reporting and harms {22}”).

#### Baseline characteristics

In addition to a measure of demographic characteristics, the Modified Trauma and Life Events Checklist (TALE) [60], a 21-item trauma screening tool for identifying clinically significant traumas in people with psychosis, will be administered at baseline.

#### Experiences of receiving and delivering therapy

The qualitative studies will be guided by semi-structured, flexible topic guides which may be revised iteratively throughout the process of data collection and analysis to respond flexibly to novel topics and themes of interest. Individual interviews will be used to explore participants’ experience of the trial and intervention, including any perceived positive and negative aspects; therapy components they have implemented into their lives; recommendations for improvement; any perceived impact on their personal recovery and mental health (with an emphasis on the hypothesised mechanisms of dissociation, negative self-beliefs, and dysfunctional patterns of voice relating) during and following TWV therapy; if and how the intervention may have contributed to these changes, and to draw patterns across participants’ experiences. A second series of qualitative interviews will explore the influence of minoritised ethnic heritage on receiving and engaging with TWV therapy, and a third focus group study will examine key themes associated with the delivery and implementation of TWV within NHS services from the perspective of trial therapists. All qualitative data will be audio-recorded with participants’ consent and transcribed verbatim for analysis.

#### Training and supervision of assessors

RAs require a bachelor level degree in psychology, or another related discipline, and experience of working in health and social care settings. All RAs received trial-specific research and clinical training from the trial manager and a lived experience expert at trial commencement and 15 months into the trial, including training on trial SOPs and all outcome measures, in order to promote GCP compliance and data quality. RAs also receive regular supervision from site PIs and weekly 60-min individual supervision from the trial manager (focussing on such areas as safeguarding, risk management, and ensuring any participant distress which may arise throughout assessments is appropriately addressed; compliance to the trial protocol, SOPs, and local NHS policy; outcome measure queries and scoring questions; eligibility queries and reviews; monitoring assessment, liaison and retention and problem-solving any issues; and maintaining personal wellbeing). In addition, they also attend monthly group supervision facilitated by the trial manager to promote reflective practice, peer connection and shared learning across sites, and attend monthly group inter-rater reliability training for the PSYRATS-AH. To aid with the latter process, assessments may be audio-recorded with participant permission to check the quality and reliability of the assessment and scores.

Both trial therapists and RAs are NHS employed members of staff and are required to keep updated with Trust-approved mandatory training. All members of trial staff complete training in GCP and are required to familiarise themselves with the trial protocol, SOPs, and relevant trial-specific manuals for their role.

Trainee clinical psychologists will conduct the qualitative interviews with participants while drawing on appropriate supervision and support from members of the trial team. The therapist focus group will be convened by an NHS worker with lived experience of voice-hearing and/or psychosis and experience of qualitative research. The qualitative researchers will meet regularly with their supervisors to support their data collection and development of the interview schedule throughout the course of the studies, as well as supporting the development of emerging thematic and conceptual outputs.

### Plans to promote participant retention and complete follow-up {18b}

Our sample size calculation allows for an attrition rate of 15%; however, this is a conservative estimate, with the attrition rate being 10% in our pilot trial. To promote retention, participants are sent a thank you card at 4 months and 11 months post-randomisation as an expression of appreciation for their contributions to the project and are additionally given £20 in acknowledgement of their time at each research assessment (£60 in total, or £40 total for participants who only receive an 8-month follow-up assessment). Participants who take part in one of the optional qualitative interview studies on their experiences of therapy will receive a further £20 for their time. All participants also receive an allocation call from a clinically qualified member of the trial team which, in addition to providing opportunities to explore their responses to allocation, allows them to be asked for their feedback on the assessment process and promote retention by refining future contact to align with their preferences.

In order to offer a person-centred assessment approach and facilitate engagement, flexibility, and participant choice, RAs receive pre-trial training delivered by both a clinician and lived experience expert which centres on minimising burden and ensuring appropriate care and encouragement throughout the assessment process. RAs follow a standardised protocol for managing distress, and participants are additionally offered a wellbeing check-in call after assessments to provide an opportunity for emotional support, as well as the Chance to offer feedback and express a preference for how they may like future follow-ups to be conducted. A 2-month window is utilised for follow-ups to maximise available outcome data and participants are contacted in advance of their follow-up due date and provided with contact options (e.g. the opportunity to conduct the assessment over the telephone or over video call instead of face to face, or meeting for shorter appointments spread across multiple occasions) to minimise burden and promote retention into the trial. The complete battery of assessment measures (as listed in section “Plans for assessment and collection of outcomes {18a}”) will be administered to all participants willing to complete them. This also applies to participants who have discontinued or withdrawn from the intervention or whose individual intervention has deviated from the intervention protocol. RAs work within an assertive outreach model, offering participant choice about the timing and location of appointments in addition to ordering outcome measures in priority, reiterating choices to decline questions/measures, and ensuring adequate breaks are taken as required. RAs discuss follow-ups at weekly trial management supervision to promote problem solving for participant retention issues that arise, and these are also discussed at site meetings and the TMG to share cross-study learning.

### Data management {19}

Participant research data are collected and stored only after the informed consent process is complete. All research assessment data are stored separately to personally identifiable data, and each participant is provided with a unique trial identification number that is written on all assessment forms and relevant datasheets and databases. A registration record linking patient identity, contact details and trial identification number is kept electronically at each site and stored securely, with access granted only to authorised users as per the delegation log. Paper data for participants (i.e. research assessment measures for baseline, 8- and 14-month follow-up) are stored securely in locked cabinets on approved NHS and University premises, whereas electronic data are stored on approved NHS or University drives. All data are kept in accordance with General Data Protection Regulation, with paper research assessment measures inputted on a web-based electronic data capture system (InferMed MACRO version 4; KCTU), which is compliant with GCP guidelines and hosted on a dedicated server within KCL.

RAs are expected to enter data onto MACRO within 1 week of the research assessment. Functionality on MACRO includes the use of range checks for specific data fields to reduce the risk of entry errors, as well as missing data codes programmed into fields for ease of analysis. To aid monitoring, the system is also programmed to flag when a missing data code is entered. Role-specific logins and training for MACRO further increases the usability and ability to monitor and audit data entry, with no data inputted or amended independently of the study site responsible for entering the data. Data entered from paper source worksheets completed at sites will also be checked against electronic data for accuracy; to date, initial site audits conducted in spring 2024 included an accuracy check of all baseline primary outcome data and all data from one mechanisms outcome measure currently available at the site. Accuracy will be further checked for 100% of the primary outcome for all baseline data across all sites. If the error rate is greater than 1% accuracy checks for all data will be triggered.

To protect the blind, a separate MACRO database is used to capture therapy attendance and AE data. Trial therapists and RAs also input data into site-specific NHS clinical records systems, including progress notes for research and/or therapy appointments and letters. A copy of the participant’s consent form is additionally uploaded onto their NHS clinical record, with researchers’ use of clinical record systems stipulated as part of the informed consent process. The statistical staff at KCTU will have access to the final trial dataset, and after the main publication the CIs, co-investigators, and trial manager will likewise gain access. In accordance with the sponsor’s Information Governance procedures, the retention period after the end of the study is 5 years for consent forms and research management documents, whereas data will be retained for 5 years after the primary publication.

### Confidentiality {27}

All personally identifiable information (PID) for participants is password protected and securely stored on NHS or University drives with access granted only to authorised members of the research team. Paper copies of assessment materials are pseudo-anonymised with a Participant Identification Number and stored in secure NHS or University premises separate to PID. Participants are informed of the security and confidentiality of their data, including the mandated limits to confidentiality in the event of researchers being provided with information that indicates the participant or another person is deemed to be at risk. Audio recordings are conducted in a manner consistent with NHS policies and procedures at the individual site, then transferred to a secure NHS drive which are accessible only to members of the research team with assigned responsibility, as per the study delegation log. Further, all qualitative interviews will be transcribed by members of the research team with any identifying information (e.g. names, places) removed.

### Plans for collection, laboratory evaluation, and storage of biological specimens for genetic or molecular analysis in this trial/future use {33}

No biological specimens will be collected as part of the trial.

## Statistical methods

### Statistical methods for primary and secondary outcomes {20a}

A detailed SAP is in preparation and will be approved by the TMG, DMEC, and TSC prior to publication on the ISRCTN registry.

We will report participant flow using the CONSORT 2018 extension for social and psychological intervention trials [70]. Assessment of recruitment, drop-out, and completeness of therapy will be summarised by descriptive statistics. The primary analyses will use the intention-to-treat population to estimate the treatment policy estimand. The intention to treat population will consist of all participants randomised, analysed in the arms to which they were randomised, regardless of treatment switching. Statisticians will be unblinded after database lock as the statistical analysis needs to account for therapist effects in the TWV arm.

To test the primary hypothesis, we will fit a linear mixed model to the repeated measures of the QPR at 8 and 14 months, with fixed effects of randomisation, time, time by randomisation interaction, site, diagnosis, and baseline QPR, and random effects for participants and therapist. The treatment policy estimand will be estimated as the adjusted between-group mean difference from the model for each timepoint separately. All hypotheses for secondary outcomes will be analysed using linear mixed models for continuous outcomes and logistic mixed models for binary outcomes.

To test treatment-effect mechanisms, mediation analysis will use parametric regression models to estimate the indirect effects of TWV on the mechanism measures and of mechanisms on primary and key secondary outcomes, if an effect is found. Results will be reported using A Guideline for Reporting Mediation Analysis (AGrEMA) guidelines [71].

### Interim analyses {21b}

No interim analysis is planned.

### Methods for additional analyses (e.g. subgroup analyses) {20b}

Any pre-specified subgroup analyses will be detailed in the SAP before analysis is undertaken.

### Methods in analysis to handle protocol non-adherence and any statistical methods to handle missing data {20c}

Maximum likelihood estimation will allow for missing outcome data under a missing-at-random assumption, conditional on the covariates in the model.

### Plans to give access to the full protocol, participant-level data, and statistical code {31c}

The final trial dataset and statistical code will be held and managed by KCTU, and reasonable requests for access to the dataset and statistical code will be considered in the first instance by the CIs and the TMG and then KCTU.

#### Qualitative analysis

All data will be analysed using Thematic Analysis in accordance with the process outlined by Braun and Clarke [72]. Thematic Analysis is a flexible qualitative method in which verbatim transcriptions are systematically and iteratively coded line-by-line, with codes compared, contrasted and amalgamated to develop detailed themes describing patterns in participants’ experiences. Negative case data (i.e. that which is contradictory to other themes) will also be sought and described where possible. Data management and analysis will be supported by Microsoft Excel software. We will take a critical realist position, and data will be coded at a manifest level (i.e. analysing only the immediate meaning of participants’ language) to produce an accessible body of coded data from which meaningful thematic representations of participants’ perspectives can be reported.

To achieve our objectives of exploring mechanisms of action for TWV, participant interviews will be analysed to investigate the mechanisms by which the intervention is perceived to operate. Here we will employ an inductive approach whereby the researchers will not impose a pre-existing theoretical framework but will seek to identify and code data that offer relevant information about how participants experience or perceive the intervention to have impacted on their mental health, voice-hearing experiences, and personal recovery, and to draw patterns across participants’ experiences.

Analysis of the interviews with therapy participants will be conducted by independent researchers who do not work on the main trial and overseen by members of the TWV team, including those with lived experience of hearing voices. The therapist focus group will be chaired by a lived experience NHS worker, then analysed by members of the trial team (EL, AB and WJ), all of whom have lived experience of hearing voices. Participants in all three qualitative studies may be approached again once an initial analysis has been completed to provide feedback, with additional member checking [73] conducted with trial staff and lived experience consultants to enhance the trustworthiness and transparency of the final analysis.

## Oversight and monitoring

### Composition of the coordinating centre and trial steering committee {5d}

GMMH NHS Foundation Trust is the study’s primary sponsor. The daily running and organisation of the trial is coordinated by a central management team comprised of the CIs, the trial manager and the clinical lead, with site management overseen by PIs. The central management team meets on a weekly basis, with an additional TMG meeting convened monthly which is attended by the central management team, PIs, and co-investigators, including a lived experience researcher. PIs additionally hold regular team meetings with the RAs and therapists at the specific site (with appropriate measures for avoiding blind breaks); the trial manager also attends these meetings according to site requirements approximately once per month to ensure central management presence and support.

The TSC is comprised of an independent chairperson, a statistician with expertise in medical statistics, a clinician with expertise in psychological interventions for SMHP, and a lived experience representative, with both CIs and the trial manager also attending as non-independent members. Prior to each meeting, all TSC members are required to report any competing interests. The TSC’s role is to provide supervision and oversight of the project on behalf of the sponsor and funder, and to ensure the project is run in accordance with GCP and UK Policy Framework for Health and Social Care standards. It convenes biannually, with meetings scheduled approximately one fortnight following the DMEC to ensure recommendations from the latter are duly considered. The funder is provided with the minutes from both TSC and DMEC meetings, along with a summary of the committee’s suggestions and any actions taken. In this regard, both the TSC and DMEC additionally reviewed internal pilot data against the a-priori progression criteria (Table 3) in order to make appropriate recommendations to the funder.

**Table 3:** Talking With Voices internal pilot progression criteria

**Threshold**	**Red**		**Amber**		**Green**	
	**%**	**(*N*)**	**%**	**(*N*)**	**%**	**(*N*)**
Trial recruitment	≤ 59	(≤ 97)	60–99	(98–162)	100	(163)
Recruitment rate per month	≤ 59	(≤ 8)	60–99	(9–14)	100	(15)
Number of sites opened	≤ 50	(≤ 2)	70	(3)	100	(4)
Proportion receiving allocated intervention	≤ 59	(≤ 48)	60–99	(49–81)	100	(82)
Proportion with complete primary outcome data	≤ 84	(≤ 25)	85–99	(26–29)	100	(30)

### Composition of the data monitoring committee, its role and reporting structure {21a}

The DMEC is comprised of a chairperson, a statistician with expertise in medical statistics, and a clinician with expertise in psychological interventions for SMHP. All above members are likewise independent of the sponsor, funder, and trial team and are required to report any competing interests prior to each meeting. DMEC meetings are further attended by both CIs, the trial manager, and the trial statistician and convene biannually with an option for dealing with any arising issues between meetings via phone or email if necessary. Meetings focus on reviewing trial progress and accruing data, including recruitment, ethical issues of consent, retention, data quality (e.g. return rates, treatment compliance), the incidence of AEs and SAEs, and any other factors that might compromise the conduct, progress, and satisfactory completion of the trial. A copy of the DMEC charter is retained by the trial manager in the ISF and the DMEC is further required to provide advice on the overall conduct and progress of the trial to the TSC.

### Adverse event reporting and harms {22}

An AE is defined as any untoward medical occurrence for a participant which may or may not be deemed related to study procedures or interventions. AEs capture a broad range of unfavourable or unintended clinical indicators, including symptom or disease, abnormal laboratory results, traffic accidents, and on some occasions reported increases in psychological distress and incidences of self-harm. Close attention is paid to AEs which have a temporal relationship to a trial procedure or intervention session in order to assess potential relatedness to the trial. Consistent with Health Research Authority (HRA) guidance for a non-CTIMP (i.e. the trial is not a Clinical Trial of an Investigational Medicinal Product), an SAE is defined as an AE that either (1) results in death, (2) is life threatening, (3) requires hospitalisation or prolongation of existing hospitalisation, (4) results in persistent or significant disability or incapacity, (5) consists of a congenital anomaly or birth defect, or (6) is another important medical event deemed serious based on clinical judgement. SAEs are not inclusive of planned hospitalisations for pre-existing conditions, unless there is also a serious deterioration in health.

(S)AE data is collected and monitored for all participants from the time of their enrolment into the study (i.e. the time at which they sign and date the study consent form). Such data is collected via spontaneous reporting from participants or their care teams to RAs or trial therapists, and via checking electronic patient records for risk updates ahead of assessment or intervention sessions. We also capture potential (S)AEs related to the study procedures and intervention in our participant feedback questionnaire given to participants at the point of trial exit. As those allocated to the intervention arm have weekly contact with a trial therapist, we would expect to see a reporting bias whereby more events are recorded for those in the therapy condition compared to those in TAU. To address this, we employ a systematic approach to identifying any SAEs not already reported to study team members via screening all participants’ electronic patient records at the point of trial exit.

In accordance with the study SOP on (S)AE reporting, members of the trial team are expected to report all (S)AEs to the trial manager within 24 h of becoming aware of the event. These are then reviewed to determine severity, intensity, causality (relatedness), and expectedness in line with HRA guidance for classification of (S)AEs for non-CTIMPs. We use Medical Dictionary for Regulatory Activities (MedDRA) System Organ Class (SOC) categories to standardise reporting of (S)AEs. In view of the study population, these are categorised under the “Psychiatric Disorders” SOC into further subcategories of self-injury (not suicide), suicidal ideation with a behavioural component, suicide, voluntary psychiatric hospital admission, involuntary psychiatric hospital admission, and other psychiatric events. In the event of uncertainty about classification, guidance is sought from the CIs. Events deemed expected for the study population include psychiatric hospital admissions, self-harm, suicidal ideation with or without a behavioural component, acting on commanding voices, and psychological distress following trial assessments and/or psychological therapy. These events have been deemed expected for the study population based on commonly occurring (S)AEs from our pilot study, and from consultation with our oversight committees (DMEC and TSC).

All (S)AEs are reported and monitored at monthly TMG meetings and at each meeting of the DMEC. The chair of the DMEC will also be informed of any SAEs that are deemed possibly or probably related to trial procedures or intervention in between meetings. It has been agreed between the TSC and DMEC committees that in the event of a pattern or significant number of related SAEs, a different procedure of independent monitoring may be agreed upon. SAEs are reported by the trial manager or CIs to the study sponsor as soon as possible (typically within 24 h) and SAEs classified as both unanticipated and related to study procedures will be reported to the REC/ HRA within 15 calendar days. Such events will also be reported to the REC/HRA immediately (and no later than two calendar days) for incidents that indicate risk of imminent death, serious injury, or serious illness, and require prompt remedial action for other participants. The AE database was made available to the sponsor for audit purposes in spring 2024 and a report of all AEs will be made available to the sponsor on request. All AEs and SAEs are entered by the trial manager onto the unblinded therapy attendance MACRO database and are reported during DMEC meetings. These processes aim to provide independent scrutiny of (S)AEs at different levels throughout the duration of the trial. Within the trial’s outcome publication, we will report details of all events classified as AEs and SAEs, regardless of expectedness or relatedness to study procedures or intervention.

### Frequency and plans for auditing trial conduct {23}

Trial audits at each site are implemented by the sponsor and delegated to the trial manager and CIs, consistent with a sponsor approved monitoring plan. Inspections focus on monitoring the ISF for its accuracy and comprehensiveness, including the completion of eligibility check forms; accuracy checks for all baseline primary outcome data and all data from at least one mechanistic outcome measure; accurate and complete recording of Changes in participant status; conduct of informed consent procedures in accordance with the protocol and GCP guidelines; accuracy of consent forms; verifying that consent is taken only by those delegated as per the delegation log; and secure, confidential storage of participant data. Trial audits of all study sites took place in Spring 2024 and are due to reoccur in Summer 2025. Auditing processes are not independent of the investigators or the sponsor.

### Plans for communicating important protocol amendments to relevant parties (e.g. trial participants, ethical committees) {25}

Amendments to the study protocol are expected to be limited throughout the duration of the trial and only made with the approval of the trial manager, CIs, and NIHR Programme Manager. Such amendments may be identified following feedback from trial team members and are discussed at the monthly TMG meeting; proposed amendments are then submitted to the sponsor and the funder for review and approval. All amendments to the protocol are reported to DMEC and TSC oversight committees and, where required, may also be raised to obtain the committees’ opinion ahead of seeking sponsor and funder approval.

The study team must complete the HRA-approved amendment toolkit for all proposed study amendments, which confirms if an amendment meets criteria for substantial or non-substantial, with further categorisation to advise on what level of approval is required from each study site. The protocol is a version-controlled document, and current and previous versions of the trial protocol are stored in the ISF. In line with NIHR guidance, the trial protocol will be made publicly available via the ISCRTN registry within 12 months of study completion.

#### Termination criteria

The sponsor may suspend or prematurely terminate the study at an individual investigation site, or the entire trial, for significant and documented reasons (e.g. when recommended by the DMEC). When instructed by relevant regulatory authorities, or if suspicion of an unacceptable risk arises, the sponsor will suspend the study while risk is assessed. The sponsor shall terminate the study if an unacceptable risk which cannot be controlled is confirmed and shall consider terminating, or suspending, the participation of a particular investigator or investigation site if serious or repeated deviations are identified. If suspension or premature termination occurs, the terminating party shall justify its decision in writing and promptly inform the other parties with whom they are in direct communication. If, for any reason, the sponsor suspends or prematurely terminates the trial at an individual investigation site, the sponsor shall inform the responsible regulatory authority as appropriate and ensure that relevant bodies are notified, either by the CIs or by the sponsor. If the suspension or premature termination was in the interest of safety, then the sponsor shall further inform all other PIs. In the case of suspension or premature termination access to, or breaking of, the blinding code would be decided by the DMEC.

#### Patient and public involvement

Given the influence of the HVM in developing the intervention, the TWV-II trial places high value on lived experience perspectives and has aimed for study implementation to be co-produced at relevant stages. In this respect, trial manager EJ has lived experience of psychosis, co-CI EL is a lived experience researcher, and two additional members of the study team (AB, WJ) likewise have experience of hearing voices and SMHP. Alongside clinically qualified colleagues, training of research staff and therapists is co-delivered by EL and/or AB, with supervision of trial therapists following the same model. A lived experience researcher also contributes to TMG meetings, and additional Patient and Public Involvement (PPI) scrutiny is ensured for study procedures by inviting a representative with lived experience to be a member of the TSC. PPI activities are monitored and recorded under relevant agenda items during TMG meetings, oversight committees, and local site meetings, with the Psychosis Research Unit’s (PRU) Service User Reference Group (SURG) providing additional consultation for relevant phases of study design and implementation, including the development of participant-facing materials.

### Dissemination plans {31a}

The study will provide evidence regarding the clinical efficacy of a novel, user-informed intervention that uses direct dialogue to reduce the impact of distressing voice hearing in a transdiagnostic population. This output is intended to address several unmet needs, including improving the efficacy and accessibility of evidence-based psychosocial interventions for adults with SMHP, developing the workforce, and responding to the NHS’s Long-Term Plan for implementing personalised, trauma-informed care as part of mental health provision. A dissemination policy is under preparation, which outlines plans for sharing the findings amongst clinicians and researchers via high-quality peer-reviewed publications. We will also make intervention manuals freely available via a web portal for clinicians to utilise in order to facilitate effective uptake, sustainability, and implementation.

Our team has a successful record of sharing study findings and will utilise existing strategies to achieve this, including workshops and conference presentations delivered to a diverse range of audiences (e.g. service-users and their families, healthcare professionals, and academics). The study findings will also be hosted on the trial’s website for further dissemination amongst participants (who are provided with a link for the latter within the “Thank You” cards provided at 4- and 11-month post-randomisation and at trial exit), the public, researchers, and clinicians. We will further generate quantitative data that may be of interest to researchers examining the efficacy of psychosocial interventions (e.g. for systematic review, meta-analyses, and individual patient data analysis), including the impact of targeting key psychological processes to minimise distressing voice hearing.

PPI consultation will additionally be utilised to ensure accessible summaries for trial participants and members of the public to be hosted on the trial’s website, and we will endeavour to embed the perspective of voice-hearers in sharing the results, including presentations delivered by team members with experience of SMHP, engaging with voluntary sector organisations like the Hearing Voices Network, and consulting with our SURG where appropriate for feedback on our dissemination strategy.

#### Authorship eligibility guidelines

Authorship eligibility for all trial-associated publications will follow the International Committee of Medical Journal Editors (ICJME) criteria for authorship. We anticipate three major classes of publications, as follows: (1) reports of the main trial outcomes, (2) reports addressing one aspect of the trial or findings in detail, (3) reports of any nested sub-studies by a subset of trial sites/staff. Proposed topics for presentation or publication will be agreed initially with the CIs and trial manager before being circulated to the TMG for approval. Prior to commencing a publication, the CIs and trial manager will review the list of research team members to consider who would meet the ICJME criteria, with group authorship considered where appropriate. We do not intend to use professional writers for any publications related to the TWV-II trial.

## Discussion

For people with diagnoses of SMHP, hearing distressing voices is a common experience [1–3] which can have a severe and longstanding impact [5–7]. Given that a relatively high number of individuals may identify trauma as a contributing factor to their voices [74–76], psychological therapies are cited as a desired form of support by many service users [77] with greater expansion of, and access to, psychological interventions for psychosis being emphasised by mental health services more generally [78, 79], including those which are specifically trauma-informed [80]. Correspondingly, TWV has the potential to improve both access and application of trauma-informed care for distressed voice-hearers, including those who have not significantly benefitted from prevailing approaches like antipsychotic medication. It additionally represents an important innovation though the active involvement of individuals with lived experience, both in the delivery of the study and development of the therapy itself. In this regard, TWV is an example of a novel intervention, informed by the survivor-led work and ethos of the HVM, which has already taken on a degree of delivery across various countries without undergoing robust evaluation. As such, the trial demonstrates the application of an approach which has engaged with the empirical framework for testing its value while still emphasising the philosophy of the HVM; both in the active involvement of investigators with lived experience, and in choosing an outcome measure that maps onto the appropriate goals of the therapy.

If shown to be efficacious, safe, and acceptable, TWV could enhance choice for service users by adding a new alternative to a range of evidence-based interventions. In this respect, there is variation within and between voice-hearers for their preferred treatment goals [74] and it is possible that specific interventions, including TWV, may have differential effects on different outcomes, thereby helping to promote informed choice in terms of engagement with, and likely benefit from, such treatments. Furthermore, TWV can be applied across transdiagnostic populations, potentially offering support to individuals who may not meet criteria for existing services and therefore struggle to access psychological interventions from the NHS. TWV’s mode of delivery may also offer scope for tailoring choice of treatment according to voice characteristics; specifically, for those who have complex, interpersonally dynamic voices, as opposed to those in which single words, short utterances and less evolved relationships are seen and for whom other approaches, such as CBTp, may be more appropriate. This, in turn, could support broader attitudinal shifts across services, wherein phenomenology is actively discussed and incorporated as part of treatment planning rather than merely assessing for the presence/absence of hallucinations to ascertain care. As such, TWV holds potential to positively impact individuals troubled by distressing voices, thereby enhancing healthcare cost-effectiveness, as well as fostering workforce development and improving clinician aptitude in delivering relational interventions to voice-hearers. It should also be noted that a foreseeable challenge to providing prompt clinical benefits is the potential lack of appropriate resources and training. However, it is intended that dissemination of a structured therapy protocol, along with related training materials, will help to facilitate effective implementation, sustainability, and scalability across various mental healthcare settings.

Taken together, the TWV-II trial will add to the evidence base for the range of psychosocial interventions that should be provided to improve outcomes for people with SMHPs, who remain amongst the most excluded groups in society. We have taken a robust approach to minimise bias, including a randomisation system hosted by a UK-registered CTU, trial-specific SOPs, pre-specification of statistical analyses, and a rigorous framework for recording and reporting adverse events. The trial is further designed to answer clinically significant hypotheses using the fewest number of participants, thereby maximising the use of resources and value for money. Specifically, it will provide evidence for the clinical efficacy of a psychological therapy, deliverable within the NHS, that is intended to reduce the impact of persistent, distressing voices amongst adults with SMHP, as well as generating data on hypothesised treatment mechanisms, thereby offering potential further improvements and refinements for future interventions which target distressing voices and a greater understanding of voice hearing itself. In this regard schizophrenia, the diagnosis with which voice hearing is most closely associated, is a significant economic burden in the UK [8], and developing evidence-based interventions to support this population may contribute to sizeable savings for the health and social care budget, in addition to reducing the personal costs for service users and their families. If the intervention is found to be significantly superior to TAU in promoting recovery and reducing the negative impact of voices without an adverse effect burden, this could have implications for the future evidence-based management of service users with similar difficulties within mental health services.

## Trial status

 V2.2 (23.06.2025) of the trial protocol has received ethical approval and at the time of publication is the current protocol. Recruitment commenced on 01 September 2023 and completed on 30 June 2025. The end of the trial is defined by the last visit to the last participant, which will occur in January 2026.

## Supplementary Information

Supplementary Material 1. Appendix A.

## Data Availability

Data and materials will be made available as required to the sponsor, REC, and DMEC for the purposes of monitoring, auditing, and review. The final trial dataset and statistical code will be held and managed by KCTU, and reasonable requests for access to the dataset and statistical code will be considered in the first instance by the CIs and then KCTU following publication of the main results paper.

## Abbreviations

AE: Adverse event
BAVQ-R: The Revised Beliefs About Voices Questionnaire
BCSS: Brief Core Schema Scale
AGrEMA: A Guideline for Reporting Mediation Analysis
CBT: Cognitive behavioural therapy
CBTp: Cognitive behavioural therapy for psychosis
CI: Chief Investigator
CMHT: Community mental health teams
CONSORT: Consolidated Standards of Reporting Trials
CTIMP: Clinical Trial of an Investigational Medicinal Product
DES-II: The Dissociative Experiences Scale-II
DMEC: Data Monitoring and Ethics Committee
GCP: Good Clinical Practice
GMMH: Greater Manchester Mental Health NHS Foundation Trust
HRA: Health Research Authority
HVM: Hearing Voices Movement
ICC: Intraclass correlation coefficient
ICMJE: International Committee of Medical Journal Editors
ISF: Investigator Site File
KCL: King’s College London
KCTU: King’s Clinical Trials Unit
MedDRA: Medical Dictionary for Regulatory Activities
MRC: Medical Research Council
NICE: National Institute for Health and Care Excellence
NIHR: National Institute for Health Research
NHS: National Health Service
PCL-5: Post Traumatic Stress Disorder PTSD Checklist for DSM-5
PI: Principal Investigator
PID: Personally identifiable information
PIS: Participant information sheet
PPI: Patient and Public Involvement
PRU: Psychosis Research Unit
PSYRATS-AH: Psychotic Symptom Rating Scales – Auditory Hallucinations Subscale
PSYRATS-MMH: Psychotic Symptoms Rating Scale – Multimodal Hallucinations
QPR: Questionnaire About the Process of Recovery
RA: Research assistant
RCT: Randomised controlled trial
RDN: Research Delivery Network
REC: Research Ethics Committee
SAE: Serious adverse event
SAP: Statistical Analysis Plan
SES: Standard effect size
SMHP: Serious mental health problems
SOC: System Organ Class
SOP: Standard operating procedure
SPIRIT: Standard Protocol Items: Recommendations for Interventional Trials
SURG: Service User Reference Group
TALE: Trauma and Life Events Checklist
TAU: Treatment as usual
TMG: Trial Management Group
TSC: Trial Steering Committee
TVAQ: Trauma Voice Associations Questionnaire
TWV: Talking With Voices
UKCRC: UK Clinical Research Collaboration
VAAS-12: Voices Acceptance and Action Scale - Brief Version

## Footnotes

1 This is determined on a case-by-case basis and is assessed on a range of factors pertaining to the perceived agency, intentionality, and embodiment of the voices; e.g. whether they are able to conduct conversations with the hearer or each other as opposed to repeating a limited repertoire of phrases, if they provide meaningful responses to questions, if they exhibit awareness of their surroundings, if they indicate autonomous responses relative to the hearer, and/or they express knowledge or perceptions independently of the hearer (81).
