# Supplementary material for: Multisite randomised controlled trial of a novel dialogical therapy in comparison to treatment as usual in adults with distressing and persistent auditory hallucinations: study protocol for the Talking With Voices (TWV-II) trial
Source: Trials. 2025 Oct 13;26:405. doi: 10.1186/s13063-025-09018-y (PMC12519869; doi:10.1186/s13063-025-09018-y)
Supplement: Supplementary file 1 — Supplementary Material 1. Appendix A. [file 13063_2025_9018_MOESM1_ESM.pdf]

CONSENT FORM

Participant ID Number: ______________

Title of Project: A novel dialogical therapy (Talking With Voices) in comparison to treatment as usual in adults with distressing and persistent auditory hallucinations: A randomised controlled trial to investigate the efficacy of a treatment strategy targeted at trauma-related mechanisms

Chief Investigators: Dr Eleanor Longden and Professor Tony Morrison

		**Please initial box**
1	I confirm that I have read and understood the information sheet dated 21.02.2025 Version Number 7 for the above study. I have had the opportunity to consider the information, ask questions and that these have been answered satisfactorily.	
2	I understand that my participation is voluntary and that I am free to withdraw at any time up to the point of my data being analysed without giving a reason and without my care or legal rights being affected.	
3	I understand that my medical records may be looked at by individuals from the research team for the purposes of the study. I give permission for these individuals to have access to my records.	
4	I understand that my data collected during the study and/or medical records may be looked at by individuals from the research team, regulatory authorities or individuals from the Trust where it is relevant to my taking part in this research or for regulatory and auditing purposes. I give permission for these individuals to have access to my data and/or records.	
5	I agree to my GP being informed of my participation in the study and, if I receive therapy, to be notified of my treatment progress.	
6	I agree to take part in the study.	

		**Please initial chosen box**	
		**Yes**	**No**
1	I agree that you may audio tape therapy sessions and research assessments as required with my permission and understand that I may have a copy of any tapes made.		
2	I agree for the information collected about me to be used to support other research in the future, and that it may be shared anonymously with other researchers.		

		Yes	No
3	I agree that if I receive the Talking with Voices therapy, I may be contacted for an optional interview about my experiences of the therapy, which would be recorded and transcribed.		

Name of participant: --

Signature: --

Date: --

Name of researcher: --

Signature: --

Date: --

Completed filing arrangements:

1. *Participant copy*
2. *Site file*
3. *Medical records*

This project is funded by the National Institute for Health Research (NIHR) Postdoctoral Fellowship Scheme (Grant Reference Number PDF-2017-10-050). The views expressed are those of the authors and not necessarily those of the NIHR, the NHS, or the Department of Health.
